# Extensive Subcutaneous Emphysema and Pneumomediastinum Following Forceps-Assisted Vaginal Delivery: A Rare Case of Postpartum Hamman’s Syndrome

**DOI:** 10.7759/cureus.119402

**Published:** 2026-11-10

**Authors:** Farhana Akter, Callum Sefton

**Affiliations:** 1 Obstetrics and Gynaecology, Lancashire Teaching Hospital Trust, Preston, GBR; 2 General Practice, Royal Shrewsbury Hospital, Telford, GBR

**Keywords:** extensive subcutaneous emphysema, hamman syndrome, postpartum complications, spontaneous pneumomediastinum (spm), vaginal delivery, valsalva maneuvre (vm)

## Abstract

Spontaneous pneumomediastinum associated with subcutaneous emphysema, also known as Hamman’s syndrome, is a rare but important complication of vaginal delivery. It is thought to result from increased intrathoracic pressure during prolonged and forceful Valsalva manoeuvres in the second stage of labour, leading to alveolar rupture and the dissection of air along the bronchovascular sheaths into the mediastinum and surrounding cervical soft tissues.

We present the case of a primiparous woman who developed extensive cervicofacial subcutaneous emphysema and pneumomediastinum following forceps-assisted vaginal delivery after a prolonged second stage of labour lasting approximately two hours. On the evening following delivery, she reported progressive facial and neck swelling, discomfort on swallowing both liquids and solids, and a sensation of chest heaviness.

Clinical examination revealed facial puffiness and palpable cervical crepitus. Despite these findings, she remained clinically stable, with no breathlessness, chest pain, or evidence of respiratory compromise. Her respiratory rate was 17 breaths per minute, and oxygen saturation was 98% on room air. Chest auscultation revealed good bilateral air entry, and the initial chest radiograph demonstrated cervical and upper mediastinal emphysema.

Contrast-enhanced computed tomography of the neck, thorax, and abdomen subsequently confirmed extensive surgical emphysema extending from the angle of the jaw throughout the cervical soft tissues into the upper chest, accompanied by widespread pneumomediastinum and associated extrapleural air around the lung apices and adjacent to the mediastinum. Importantly, there was no definite pneumothorax, oesophageal leak, tracheal wall injury, or underlying pulmonary abnormality.

Following assessment by the obstetric and surgical teams, conservative management with close clinical observation and supportive care was recommended, as no surgical intervention was indicated. Her facial and neck swelling improved significantly over the subsequent days, without evidence of respiratory deterioration.

This case highlights the importance of recognising postpartum Hamman's syndrome in women presenting with cervicofacial swelling and subcutaneous crepitus following vaginal delivery. Despite extensive radiological abnormalities, the patient remained clinically stable and improved with conservative management. Appropriate imaging and multidisciplinary assessment are important for identifying associated complications and guiding individualised treatment. As this is a single case report, the findings should not be generalised to all patients with postpartum pneumomediastinum.

## Introduction

Spontaneous pneumomediastinum, also known as Hamman’s syndrome, is a rare complication of childbirth characterised by the presence of free air within the mediastinum, which may be accompanied by subcutaneous emphysema involving the neck, face, and upper chest [1-3]. Although usually self-limiting, its occurrence during labour or the immediate postpartum period can present a diagnostic challenge because the symptoms may resemble those of more serious cardiopulmonary conditions.

Spontaneous pneumomediastinum is generally associated with a sudden rise in intra-alveolar pressure, particularly during forceful Valsalva manoeuvres [4,5]. The underlying pathophysiological process is classically explained by the Macklin effect, in which rupture of peripheral alveoli permits air to dissect along the bronchovascular sheaths into the mediastinum [6]. Repeated Valsalva manoeuvres and prolonged maternal pushing during the second stage of labour may therefore precipitate this condition.

Clinical manifestations may include retrosternal chest discomfort, dyspnoea, facial or cervical swelling, dysphagia, and palpable subcutaneous crepitus [1-3]. Chest radiography is often the initial investigation, while computed tomography (CT) can more accurately demonstrate the extent of pneumomediastinum and associated soft-tissue emphysema [5]. Careful assessment is required to exclude potentially serious causes of postpartum chest symptoms, including pneumothorax, pulmonary embolism, and oesophageal or tracheobronchial injury [2,5]. Pleural disorders, including pneumothorax, are also recognised considerations during pregnancy and the peripartum period [7]. Hamman’s classic description of mediastinal emphysema contributed to the terminology subsequently associated with this syndrome [8].

We report a case of extensive cervicofacial subcutaneous emphysema and pneumomediastinum in a 31-year-old primiparous woman following forceps-assisted vaginal delivery for a delayed second stage of labour. Although postpartum Hamman's syndrome has previously been reported following both instrumental and non-instrumental vaginal delivery, the present case is notable for extensive cervicofacial subcutaneous emphysema and extrapleural air despite preserved respiratory stability. This report highlights the importance of recognising this uncommon postpartum complication following forceps-assisted delivery, evaluating potentially serious alternative diagnoses, and considering conservative management in appropriately assessed, clinically stable patients.

## Case presentation

A 31-year-old primiparous woman at term was admitted in spontaneous labour and subsequently underwent forceps-assisted vaginal delivery because of a delayed second stage of labour lasting approximately two hours. A live male infant was delivered. An episiotomy was performed and sutured, with an estimated blood loss of 500 mL. Intravenous antibiotics were administered following instrumental delivery. Approximately 11 hours postpartum, the patient developed progressive swelling of the face and neck, associated with discomfort on swallowing and a sensation of chest heaviness. She did not report breathlessness and showed no evidence of respiratory distress.

On clinical assessment, the patient was haemodynamically stable, with an oxygen saturation of 98% on room air. Examination revealed visible cervicofacial swelling with palpable crepitus over the neck, raising suspicion of subcutaneous emphysema.

An initial chest radiograph, obtained on the evening of symptom onset approximately 11 hours postpartum, demonstrated left-sided pneumomediastinum and bilateral cervical subcutaneous emphysema, with a possible tiny left apical pneumothorax (Figure 1). Following surgical assessment, contrast-enhanced computed tomography (CT) was recommended to evaluate the extent of the emphysema and investigate possible aerodigestive injury. The examination was initially delayed while concerns regarding radiation exposure and contrast administration during the postpartum period were discussed. CT was subsequently performed approximately two days after delivery.

**Figure 1:**
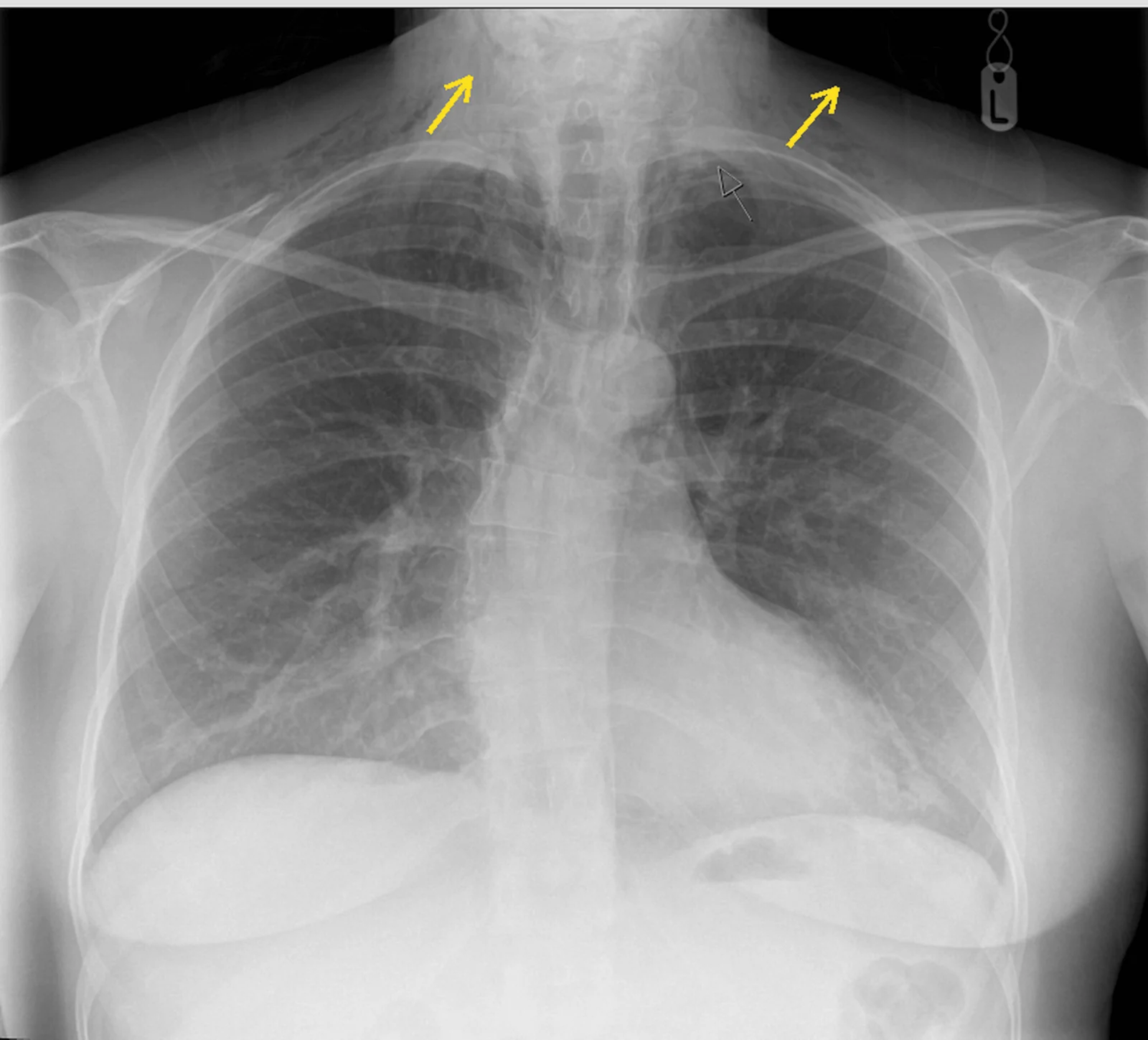
Chest radiograph demonstrating cervical subcutaneous emphysema and pneumomediastinum Chest radiograph showing bilateral cervical subcutaneous emphysema (yellow arrows) with associated left-sided pneumomediastinum.

CT demonstrated extensive subcutaneous emphysema extending from the angle of the jaw through the cervical soft tissues into the upper chest (Figure 2), together with widespread pneumomediastinum and extrapleural air. An additional axial image from the same CT examination demonstrated air tracking through the cervical and upper mediastinal tissue planes at the thoracic inlet (Figure 3). There was no definite pneumothorax, oesophageal leak, tracheal wall injury, or underlying pulmonary abnormality. The CT findings, together with the patient's preserved clinical stability, supported a diagnosis of spontaneous postpartum pneumomediastinum.

**Figure 2:**
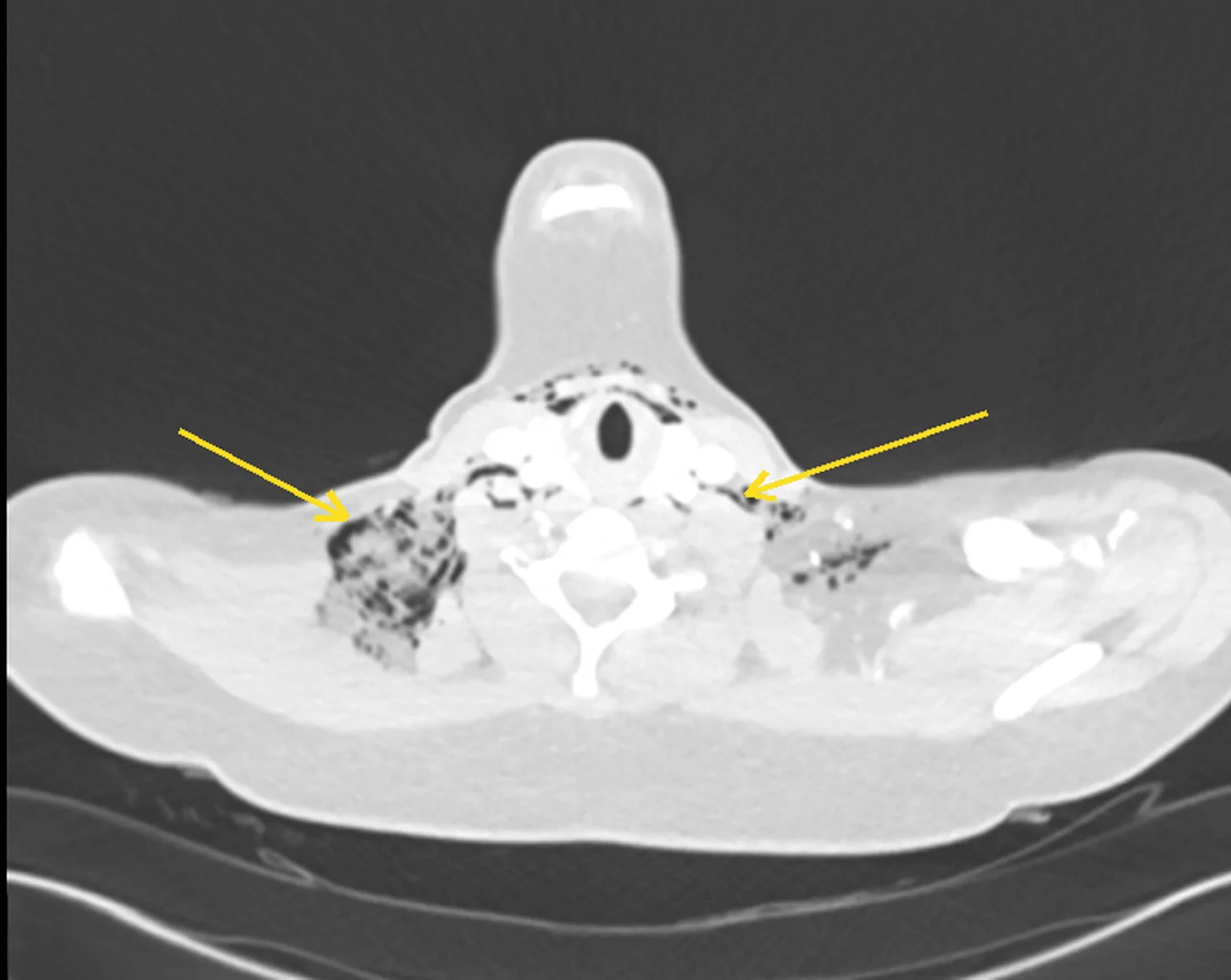
Axial computed tomography demonstrating extensive cervical subcutaneous emphysema Axial contrast-enhanced computed tomography (CT) image at the lower cervical level demonstrating extensive subcutaneous emphysema involving the cervical soft tissues bilaterally, more prominent on the right. Yellow arrows indicate areas of subcutaneous air.

**Figure 3:**
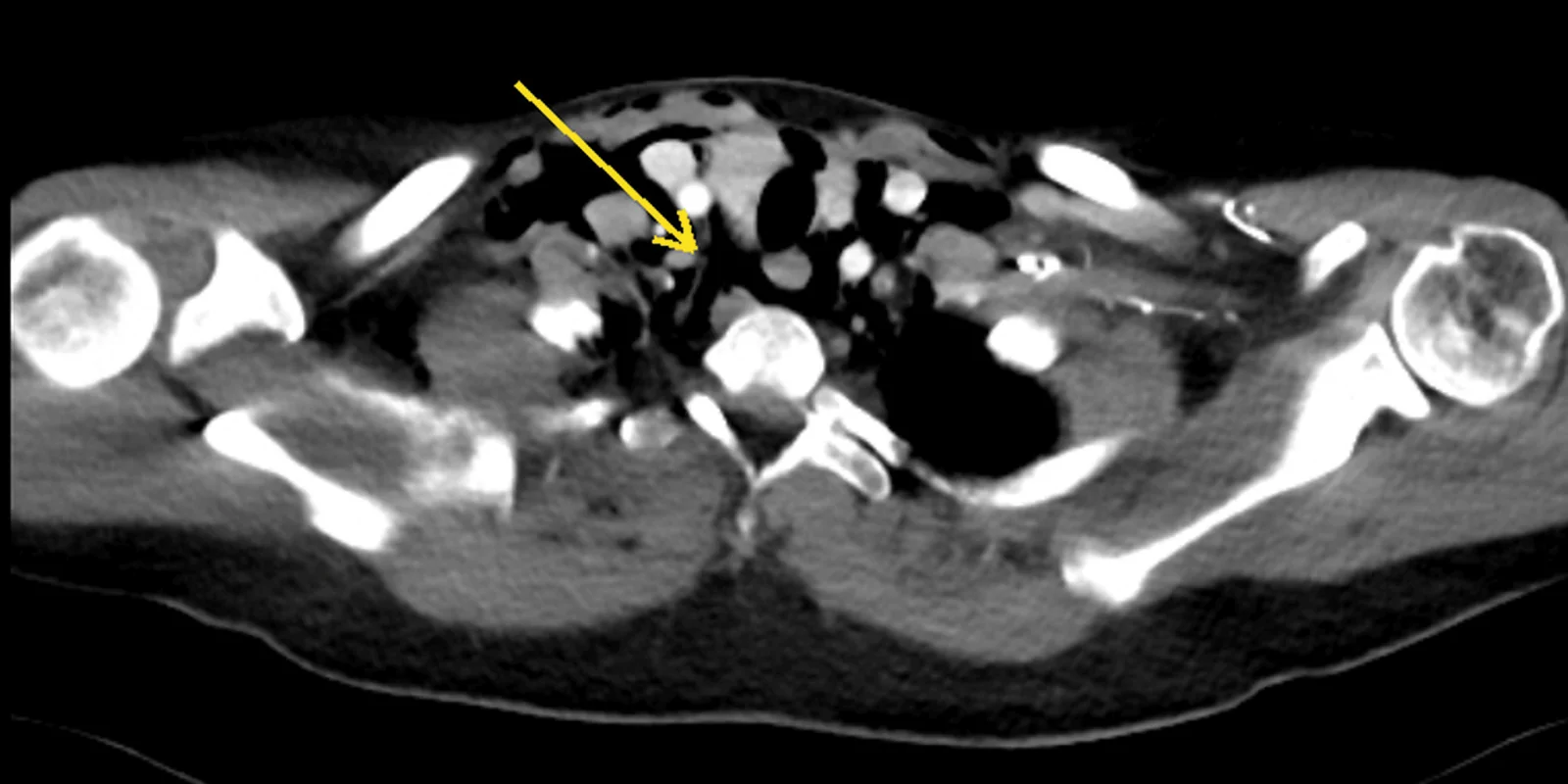
Axial computed tomography demonstrating air tracking at the thoracic inlet Axial contrast-enhanced computed tomography (CT) image at the level of the thoracic inlet demonstrating abnormal air tracking within the cervical and upper mediastinal soft tissue planes. The arrow highlights an area of extraluminal air.

The patient was reviewed by the obstetric and surgical teams. Given her stable clinical condition, preserved oxygen saturation, and absence of CT findings suggesting major aerodigestive injury, conservative management with clinical observation and supportive care was recommended. Oral intake was gradually reintroduced as tolerated. During subsequent postnatal assessments, her facial and cervical swelling decreased substantially, swallowing discomfort improved, and she remained clinically stable without respiratory compromise. By postpartum day three, she was tolerating food and fluids, mobilising independently, and reporting marked symptomatic improvement. Following review of the CT findings, the surgical team advised that no surgical intervention or routine surgical follow-up was required. She was discharged home with her baby on postpartum day three following satisfactory clinical improvement and obstetric assessment.

## Discussion

Postpartum spontaneous pneumomediastinum, also known as Hamman's syndrome, is an uncommon complication of childbirth that may be accompanied by extensive subcutaneous emphysema. Although the condition is generally self-limiting, its presentation can be alarming and may resemble more serious cardiopulmonary emergencies. Previous reports have described postpartum pneumomediastinum following both spontaneous and instrumental vaginal deliveries, with prolonged maternal pushing considered a possible contributing factor [1-3].

The pathophysiology is commonly explained by the Macklin effect, in which increased intra-alveolar pressure leads to rupture of peripheral alveoli, allowing air to dissect along the bronchovascular sheaths into the mediastinum [6]. From the mediastinum, air may extend along fascial planes into the cervical and subcutaneous tissues, producing swelling and palpable crepitus. During the second stage of labour, repeated Valsalva manoeuvres can generate substantial intrathoracic pressure changes, potentially precipitating this process [2,6]. In the present case, the prolonged second stage of labour and forceps-assisted delivery may have contributed to the development of pneumomediastinum, although a direct causal relationship cannot be established.

The clinical presentation of postpartum Hamman's syndrome varies considerably. Commonly reported symptoms include chest pain, dyspnoea, neck swelling, facial swelling, dysphagia, and subcutaneous crepitus [1-3]. In our patient, the predominant features were progressive facial and cervical swelling, discomfort on swallowing, and chest heaviness. Despite extensive subcutaneous emphysema and pneumomediastinum, she remained haemodynamically stable, with an oxygen saturation of 98% on room air and no significant respiratory compromise. This demonstrates that extensive radiological abnormalities may be present even in a clinically stable patient.

The differential diagnosis of acute chest symptoms in the postpartum period includes pulmonary embolism, pneumothorax, oesophageal perforation, tracheobronchial injury, and other potentially life-threatening cardiopulmonary conditions [2,5]. Clinical examination and appropriate imaging are therefore essential. Chest radiography is a useful initial investigation, while computed tomography provides more detailed assessment of the extent and distribution of mediastinal and subcutaneous air and may help identify associated complications [5]. In this case, CT demonstrated widespread pneumomediastinum, extensive cervical and upper chest subcutaneous emphysema, and extrapleural air, without definite pneumothorax or evidence of major aerodigestive injury. The CT examination was undertaken after an initial delay related to discussions about radiation exposure and contrast administration in the postpartum period. Although no radiological evidence of oesophageal or tracheal injury was identified, the diagnosis was supported by the absence of clinical deterioration and subsequent improvement under observation. No additional endoscopic examination or contrast swallow study was documented; therefore, the exclusion of aerodigestive injury was based on the available CT findings and clinical assessment rather than direct endoscopic confirmation.

Comparison with previously published cases provides useful clinical context. Molvi et al. described pneumomediastinum and subcutaneous emphysema following non-instrumental vaginal delivery, with improvement following conservative management [1]. Revicky et al. highlighted the importance of recognising postpartum pneumomediastinum as an uncommon cause of chest pain [2]. Kuruba and Hla reported Hamman's syndrome following forceps-assisted vaginal delivery, demonstrating that the condition can also occur in association with instrumental delivery [3]. Our case adds to this literature by demonstrating particularly extensive cervicofacial emphysema and extrapleural air despite preserved respiratory function.

The distinguishing clinical features of the present case were the extensive cervicofacial subcutaneous emphysema and extrapleural air following forceps-assisted vaginal delivery, together with the patient's preserved respiratory function despite marked radiological abnormalities. Symptoms developed approximately 11 hours postpartum, highlighting the importance of recognising delayed presentations. The educational value of this case lies in the need for careful clinical assessment, appropriate imaging, and exclusion of potentially serious alternative causes before proceeding with conservative management.

Management of uncomplicated spontaneous pneumomediastinum is generally conservative and includes clinical observation, symptomatic treatment, and assessment for underlying causes or associated complications [5]. Supplemental oxygen has been described in the literature, although the evidence supporting its routine use in patients who maintain normal oxygen saturation is limited [1,5]. In the present case, the patient remained clinically stable and was managed conservatively following obstetric and surgical assessment. Her symptoms improved significantly without the need for surgical intervention.

Although postpartum Hamman's syndrome is usually associated with a favourable outcome, early recognition remains important because its symptoms overlap with those of serious postpartum conditions. This case emphasises the value of recognising palpable cervical crepitus, obtaining appropriate radiological investigations, and using multidisciplinary assessment to guide management. Clinicians should consider spontaneous pneumomediastinum in women presenting with facial or neck swelling and chest discomfort following vaginal delivery, particularly after a prolonged second stage of labour.

## Conclusions

Postpartum Hamman's syndrome is an uncommon complication of vaginal delivery that should be considered in women presenting with acute facial or cervical swelling, chest discomfort, or palpable subcutaneous crepitus following childbirth. As its presentation may resemble potentially serious cardiopulmonary or aerodigestive conditions, careful clinical assessment and appropriate imaging are important.

This case highlights extensive cervicofacial subcutaneous emphysema and pneumomediastinum following forceps-assisted vaginal delivery for a delayed second stage of labour. Although similar cases following instrumental delivery have previously been reported, the marked extent of subcutaneous emphysema and extrapleural air despite preserved respiratory function provides an additional clinical illustration of this rare condition. The patient improved with conservative management following multidisciplinary assessment and was discharged on postpartum day three. However, the findings of a single case report cannot establish a general management strategy, and treatment decisions should be individualised according to clinical stability, imaging findings, and the exclusion of serious underlying complications.
